# GHET1 acts as a prognostic indicator and functions as an oncogenic lncRNA in cervical cancer

**DOI:** 10.1042/BSR20182506

**Published:** 2019-04-30

**Authors:** Qunchang Zhang, Yongtao Zhang, Ying Wang

**Affiliations:** 1 Department of Gynaecology and Obstetrics, Xian XD Group Hospital, Xi’an 710077, Shanxi, China; 2 Department of Gynaecology and Obstetrics, Affiliated Huxi Hospital of Jining Medical College, Shanxian County, Heze 274300, Shandong, China; 3 Department of Gynaecology, Maternal and Child Health Hospital of Tangshan, Tangshang 063000, Hebei, China

**Keywords:** biomarker, cervical cancer, GHET1, lncRNA

## Abstract

Gastric carcinoma proliferation enhancing transcript 1 (GHET1) has been suggested to serve as a promising oncogenic lncRNA in various types of human cancer. However, the role of GHET1 remained unknown in cervical cancer. In our study, we found GHET1 expression was markedly elevated in cervical cancer tissue specimens and cell lines compared with adjacent normal cervical tissue specimens and human normal cervical cell line, respectively. Then, we found high expression of GHET1 is a useful biomarker to discriminate cervical cancer tissues from non-tumorous tissues, and associated with advanced clinical stage, lymph node metastasis, distant metastasis and poor histological grade in cervical cancer patients. The survival analysis showed high GHET1 expression was an independent unfavorable prognostic factor in cervical cancer patients. Knockdown of GHET1 expression markedly inhibits cervical cancer cell proliferation, migration, and invasion. The loss-of-function study indicated knockdown of GHET1 expression markedly inhibits cervical cancer cell proliferation, migration, and invasion. In conclusion, GHET1 acts as an oncogenic lncRNA in cervical cancer.

## Introduction

Cervical cancer has been reported to be fourth most common cancer and the fourth most prominent cause of cancer-related mortality in females worldwide [1]. According to global cancer statistics, there are 569,847 new cervical cancer cases and 311,365 cervical cancer deaths in 2018 [1]. In United States, an obviously decreasing incidence and mortality trend of cervical cancer was observed owing to HPV vaccination and cervical cancer screening [2,3]. On the contrary, a significantly increasing incidence and mortality trend for cervical cancer was observed in China, which caused great threat for Chinese women [4,5]. Surgery, radiotherapy, and chemotherapy remain the major treatment methods for cervical cancer patients due to lack of effective molecular targetted therapy [6,7]. Up to now, there was still a lack of effective biomarker for early diagnosis and prognosis prediction in cervical cancer. Therefore, it is of great importance to investigate the molecular mechanisms underlying the progression and development of cervical cancer for searching novel diagnostic/prognostic biomarkers or developing novel molecular targetted therapy.

LncRNA gastric carcinoma proliferation enhancing transcript 1 (GHET1) is located at human chromosome 7q36.1 and has a length of 1913 bp [8]. GHET1 was considered as a promising oncogenic lncRNA owing to high stability, efficiency, and specificity in various types of human cancer [9]. Originally, GHET1 was found to be overexpressed in gastric cancer and involved in gastric cancer cell proliferation *in vitro* and *in vivo* [10]. Subsequently, the oncogenic role of GHET1 was suggested in lung cancer [11,12], hepatocellular carcinoma [13,14], breast cancer [15], colorectal cancer [16], esophageal squamous cell carcinoma [17], pancreatic cancer [18], bladder cancer [19], head and neck cancer [20], osteosarcoma [21], and glioma [22]. However, the clinical significance of GHET1 and the related biological function in cervical cancer have not been reported at present. Therefore, we measured the GHET1 expression in cervical cancer tissue samples and analyzed the relationship between GHET1 expression and clinicopathological characteristics for estimating the clinical significance of GHET1 in cervical cancer. Moreover, we conducted loss-of-function study for assessing the effect of GHET1 expression on GHET1 expression cell proliferation, migration, and invasion.

## Materials and methods

### Tissue specimens

A total of 94 fresh cervical cancer tissue specimens and 47 fresh frozen adjacent normal tissue specimens were obtained from Xian XD Group Hospital, Affiliated Huxi Hospital of Jining Medical College or Maternal and Child Health Hospital of Tangshan. Diagnosis of each tissue specimen was confirmed by two experienced pathologist, and all patients did not receive any antitumor treatment before surgery or biopsy. All tissue specimens were frozen in liquid nitrogen immediately after collection and stored at −80°C The project protocol was approved by the Ethic Committee of Xian XD Group Hospital, Affiliated Huxi Hospital of Jining Medical College or Maternal and Child Health Hospital of Tangshan, and the written informed consent of each case was collected.

### Cell lines

A human normal cervical cell line (Ect1/E6E7) and four human cervical cancer cell lines (SiHa, C-33A, HeLa, and CaSki) were the Cell Bank of Chinese Academy of Sciences (Shanghai, China). All cells were cultured in DMEM (Gibco, Carlsbad, CA, U.S.A.) supplemented with 10 % fetal bovine serum (FBS, Gibco, Carlsbad, CA, U.S.A.) at 37°C in a humidified incubator with 5% CO_2_.

### Quantitative real time PCR

Total RNA from tissues or cells was extracted using TRIzol (Invitrogen, Carlsbad, CA, U.S.A.), and cDNA was reversely transcribed from total RNA through using PrimeScript RT reagent Kit (TaKaRa, Dalian, China). Quantitative PCR was performed by using a standard protocol from SYBR Premix Ex Taq (TaKaRa, Dalian, China) at Applied Biosystems 7300 Real-Time PCR System (Applied Biosystems, Foster City, CA, U.S.A.). Specific primers were synthetized by Takara (Dalian, China) and were shown as follows: GHET1, forward primer, 5′-CCCCACAAATGAAGACACT-3′ and reverse primer 5′-TTCCCAACACCCTATAAGAT-3′; GAPDH, forward primer, 5′-AAGGTGAAGGTCGGAGTCAA-3′ and reverse primer 5′-AATGAAGGGGTCATTGATGGGAPDH-3′. GAPDH acted as an internal control for estimating GHET1 expression.

### siRNA transfection

For reducing GHET1 expression, siRNAs targetting GHET1 (si-GHET1#1: CGGCAGGCATTAGAGATGAACAGCA, si-GHET1#2 GAGAAAUAGUCUGUGUUGCCCUGAA, and si-GHET1#3 CAGCCGGAUACAGAGUGAAUAGUUA) and negative control (si-NC) was synthesized by GenePharma Co. Ltd. (Shanghai, China). Cervical cancer cells were seeded 24 h before transfection, and si-GHET1 or si-NC was transfected into cervical cancer cells using Lipofectamine RNAiMAX Transfection Reagent (Invitrogen, Carlsbad, CA, U.S.A.) based on the manufacturer’s instructions. The transfection efficiency was checked at 48 h after transfection.

### Cell proliferation assay

Cell Counting Kit-8 (CCK-8 kit, Dojindo, Tokyo, Japan) was used for evaluating the cell proliferation ablility. Transfected cervical cancer cells were seeded into 96-well plates with a density of 3 × 10^3^/well and cultured for 24, 48, 72, and 96 h. Then, 10 μl CCK-8 reagent was added into each well and incubated at 37°C for 2 h. The absorbance of each well at 450 nm was detected to evaluate the relative cell viability at a microplate autoreader.

### Transwell migration and invasion assays

Transwell migration and invasion assays were performed for assessing cell migration and invasion ability by using transwell chambers (8-μm pore size Corning Costar, Franklin Lakes, NJ, U.S.A.). For invasion assay *in vitro*, the upper chamber was precoated with Matrigel (BD Biosciences, San Jose, CA, U.S.A.). For migration assay *in vitro*, the upper chamber did not precoated with Matrigel. Briefly, cervical cancer cells with serum-free medium were seeded into the upper chamber, and medium with 10% FBS was added into the lower chamber. After 24 h, the cells on the lower surface were fixed with 20% methanol and stained with 0.1% Crystal violet. The number of migrated/invasive cells was counted in five randomly selected fields under a microscope.

### Statistical analysis

SPSS 22.0 statistical software (IBM Corp., Armonk, NY, U.S.A.) was used for all statistical analyses. Student’s *t* test was employed to analyze the differences between two groups. The χ^2^ test was used to assess the relationship between GHET1 expression and clinicopathological features of cervical cancer patients. Log-rank test and Cox proportional hazards regression model were utilized to assess the survival data. The *P* values less than 0.05 were considered statistically significant.

## Results

### The expression of GHET1 in cervical cancer tissues and cells

**Figure 1:**
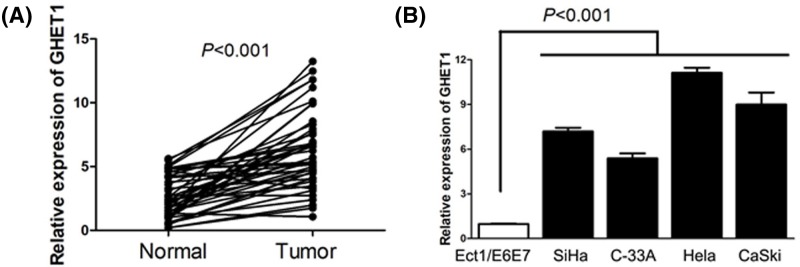
The expression of GHET1 in cervical cancer tissues and cells (**A**) GHET1 expression was markedly elevated in cervical cancer tissue specimens compared with adjacent normal cervical tissue specimens. (**B**) GHET1 expression in four cervical cancer cell lines (HeLa, C-33A, CaSki, and SiHa) was obviously increased in comparison with human normal cervical cell line (Ect1/E6E7).

To identify the expression status of GHET1 in cervical cance, GHET1 expression levels were detected by qRT-PCR in cervical cancer tissues and cell lines, and corresponding normal tissues and cell lines. We observed that GHET1 expression was markedly elevated in cervical cancer tissue specimens compared with adjacent normal cervical tissue specimens (*P*<0.001, Figure 1A). In addition, we also observed that GHET1 expression in four cervical cancer cell lines (SiHa, C-33A, HeLa, and CaSki) was obviously increased in comparison with human normal cervical cell line (Ect1/E6E7) (*P*<0.001, Figure 1B).

### The clinical significance of GHET1 in cervical cancer

**Figure 2:**
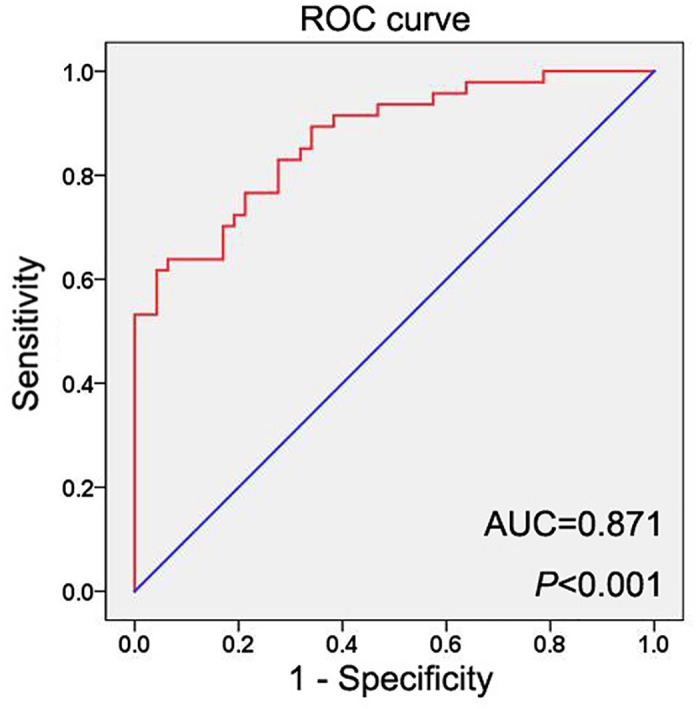
The ROC curve of GHET1 The diagnostic value of GHET1 in cervical cancer was analyzed by ROC curve analysis with AUC = 0.871.

Due to high levels of GHET1 in cervical cancer tissues and cells, GHET1 might be a useful marker to identify cervical cancer tissues from non-tumorous tissues. We drew receiver operating characteristic (ROC) curve, and the results suggested that the area under the curve (AUC) was 0.874 and 95% CI of 0.802–0.940 (*P*<0.001, Figure 2). Furthermore, we analyzed the correlations between GHET1 expression and clinicopathological parameters for estimating the clinical value of GHET1 in cervical cancer patients. As shown in Table 1, high expression of GHET1 was associated with advanced clinical stage (I–IIA vs IIB–IV, *P*<0.001), lymph node metastasis (absent vs present, *P*<0.001), distant metastasis (absent vs present vs, *P*=0.010) and poor histological grade (well vs moderately/poorly, *P*=0.003). However, we did not observe any correlation between GHET1 expression and other clinicopathological parameters including age, tumor size, and histological type.

**Table 1:** Relationships between GHET1 expression and clinicopathological parameters in cervical cancer

Parameters	n	GHET1		*P*
		High expression	Low expression	
Age(y)				
≤50	43	24 (55.8)	19 (44.2)	0.301
>50	51	23 (45.1)	28 (54.9)	
Clinical stage				
I–IIA	40	11 (27.5)	29 (72.9)	<0.001
IIB–IV	54	36 (66.7)	18 (33.3)	
Tumor size (cm)				
≤4	56	25 (44.6)	31 (55.4)	0.207
>4	38	22 (57.9)	16 (42.1)	
Lymph node metastasis				
Absent	52	17 (32.7)	35 (67.3)	<0.001
Present	42	30 (71.4)	12 (28.6)	
Distant metastasis				
Absent	86	39 (45.3)	47 (54.7)	0.010
Present	8	8 (100)	0 (0)	
Histological type				
Adenocarcinoma	9	4 (44.4)	5 (55.6)	1.000
Squamous cell carcinoma	85	43 (50.6)	42 (49.4)	
Histological grade				
Well	36	11 (30.6)	25 (69.4)	0.003
Moderately/poorly	58	36 (62.1)	22 (37.9)	

### The prognostic significance of GHET1 in cervical cancer

**Figure 3:**
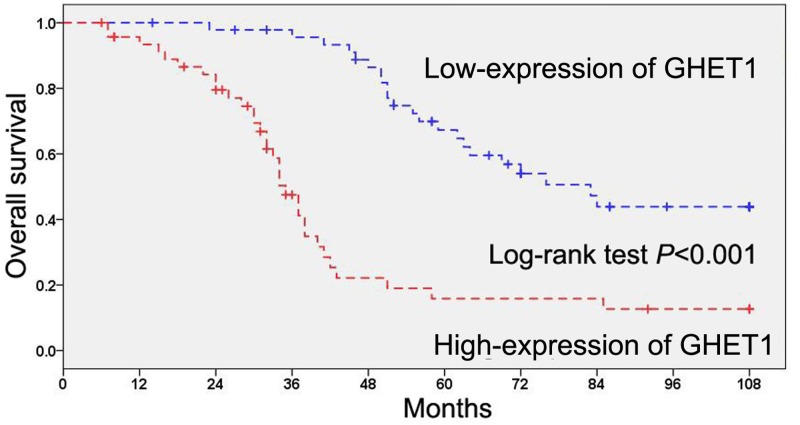
Kaplan-Meier survival curve according to GHET1 expression in cervical cancer patients The log-rank test was utilized to analyze the correlation between GHET1 expression and overall survival in cervical cancer patients.

For assessing the prognostic significance of GHET1 in cervical cancer patients, Kaplan–Meier method and log-rank test were utilized to analyze the correlation between GHET1 expression and overall survival, and indicated that cervical cancer patients with high expression of GHET1 had a worse overall survival time than patients with low expression of GHET1 (*P*<0.001, Figure 3). In addition, the univariate Cox regression analysis suggested that advanced clinical stage (*P*=0.012), lymph node metastasis (*P*=0.001), distant metastasis (*P*=0.010), poor histological grade (*P*=0.012), and high GHET1 expression (*P*<0.001) were poor prognostic factors for overall survival in patients with cervical cancer (Table 2). Then, high GHET1 expression was identified as an independent unfavorable prognostic factor in cervical cancer patients through multivariate Cox regression analysis (*P*=0.001, Table 2).

**Table 2:** Summary of univariate and multivariate Cox regression analysis of overall survival in cervical cancer

Parameter	Univariate analysis			Multivariate analysis		
	*P*	HR	95% CI	*P*	HR	95% CI
Age (y)						
(≤ 50 vs >50)	0.308	1.329	0.770–2.293			
Clinical stage						
(I–IIA vs IIB–IV)	0.012	2.038	1.170–3.548	0.697	1.217	0.453–3.271
Tumor size (cm)						
(≤ 4 vs >4)	0.860	1.050	0.612–1.801			
Lymph node metastasis						
(Absent vs present)	0.001	2.706	1.524–4.804	0.844	1.115	0.376–3.304
Distant metastasis						
(Absent vs present)	0.010	2.953	1.290–6.760	0.928	1.042	0.426–2.548
Histological type						
(Squamous cell carcinoma vs adenocarcinoma)	0.575	0.796	0.359–1.767			
Histological grade						
(Well vs moderately/poorly)	0.012	2.103	1.179–3.749	0.161	1.574	0.835–2.967
GHET1 expression						
(Low vs high )	<0.001	4.141	2.356–7.277	0.001	3.501	1.825–6.715

HR, hazard ratio.

### The biological function of GHET1 in cervical cancer

**Figure 4:**
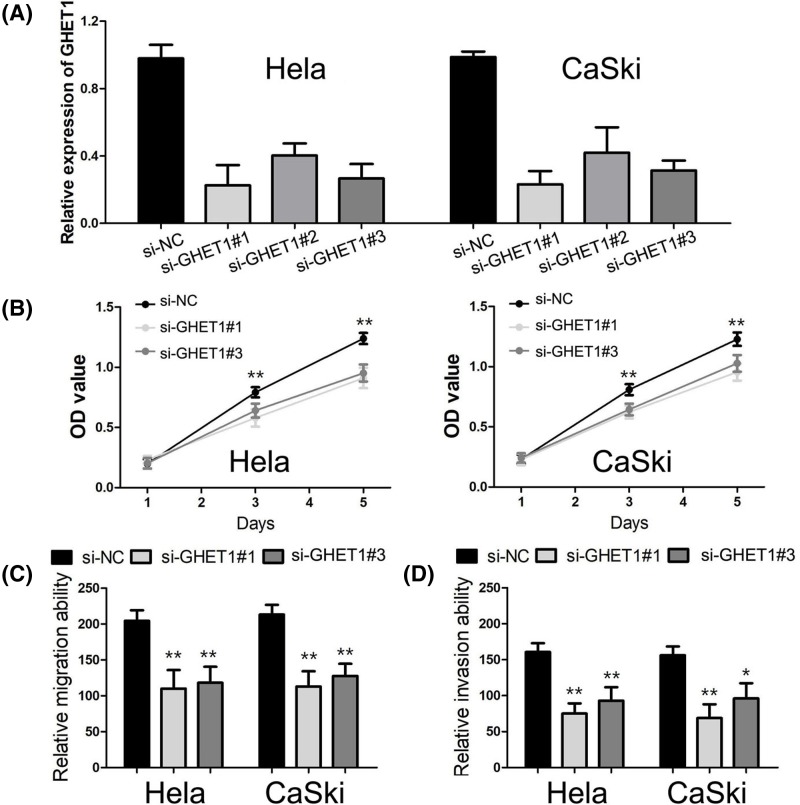
The biological function of GHET1 in cervical cancer (**A**) The qRT-PCR was conducted to check the knockdown efficiency. (**B**) The CCK-8 assay was used to assess the influence of GHET1 on cervical cancer cell proliferation. (**C**) Transwell migration assay was performed to estimate the effect of GHET1 on cervical cancer cell migration. (**D**) Transwell invasion assay was conducted to assess the impact of GHET1 on cervical cancer cell invasion. (vs si-NC; *P<0.01; **P<0.001).

Because GHET1 expression was relative high in HeLa and CaSki cells amongst four cervical cancer cell lines, we chose HeLa and CaSki cells for following loss-of-function study. To investigate the biological function of GHET1 in cervical cancer, si-GHET1 was transfected into HeLa and CaSki cells and qRT-PCR was conducted to check the knockdown efficiency (Figure 4A). We executed CCK-8 assay to assess the influence of GHET1 on cell proliferation and found that proliferation activity was obviously suppressed in cervical cancer cells transfected with si-GHET1 (*P*<0.001, Figure 4B). Moreover, transwell migration and invasion assays were performed to estimate the effect of GHET1 on cervical cancer cell migration and invasion. The results showed the migratory and invasive capabilities of cervical cancer cells were dramatically decreased after transfecting with si-GHET1 (*P*<0.001, Figure 4C,D).

## Discussion

GHET1 is a kind of intergenic lncRNA and located at human chromosome 7q36.1 [23]. Originally, Yang et al. identified high level of GHET1 expression in gastric cancer tissues [10]. Then, Huang et al. also found that GHET1 expression was increased in gastric cancer tissues and cell lines compared with adjacent tumor tissues and human gastric epithelial cell line, respectively [24]. Afterward, the role of GHET1 in carcinogenesis has caused a great deal of attention. The overexpression of GHET1 was observed in most types of human cancer such as lung cancer [11,12], hepatocellular carcinoma [13,14], breast cancer [15], colorectal cancer [16], esophageal squamous cell carcinoma [17], pancreatic cancer [18], bladder cancer [19], head and neck cancer [20], osteosarcoma [21], and glioma [22]. However, there was no report about the expression of GHET1 in cervical cancer. In our study, we also found that GHET1 expression was markedly elevated in cervical cancer tissue specimens and cell lines compared with adjacent normal cervical tissue specimens and human normal cervical cell line, respectively. Furthermore, we investigated the diagnostic and clinical significance of GHET1 in cervical cancer. We drew ROC curve and found that GHET1 is a useful biomarker to discriminate cervical cancer tissues from non-tumorous tissues. Moreover, we found high expression of GHET1 was associated with advanced clinical stage, lymph node metastasis, distant metastasis, and poor histological grade in cervical cancer patients. In gastric cancer, GHET1 overexpression was also found to be associated with large tumor size, tumor invasion, and multidrug resistance [10,25]. In addition, Shen et al. and Guan et al. conformably suggested that high levels of GHET1 expression were correlated with lymph node metastasis and advanced TNM stage in patients with non-small cell lung cancer [11,12]. In hepatocellular carcinoma patients, Jin et al. reported that GHET1 overexpression was associated with the large tumor size, vascular invasion, cirrhosis, and high edmindson grade [14]. Besides, Rui et al. indicated that high levels of GHET1 were associated with advanced clinical stage, larger tumor size, and present lymph node metastasis in breast cancer patients [15]. Li et al. also observed that high expression of GHET1 was related with big tumor size, advanced tumor stage, and lymph node status in bladder cancer patients [19]. Liu et al. and Liu et al. and similarly found that GHET1 overexpression was correlated with later TNM stage and lymph node metastasis in esophageal squamous cell carcinoma patients [17] and head and neck cancer patients [20], respectively. Additionally, GHET1 expression was also suggested to be related with clinical progression in pancreatic cancer [18] and osteosarcoma [21].

The prognostic value of GHET1 was investigated in seven kinds of human cancer in gastric cancer [10], lung cancer [11,12], hepatocellular carcinoma [14], breast cancer [15], esophageal squamous cell carcinoma [17], bladder cancer, [19] and head and neck cancer [20]. The relationship between GHET1 expression and prognosis in cervical cancer patient was still unknown. In our research, we found that cervical cancer patients with high expression of GHET1 had a worse overall survival time than patients with low expression of GHET1 through Kaplan–Meier method and log-rank test, and high GHET1 expression was identified as an independent unfavorable prognostic factor in cervical cancer patients through univariate and multivariate Cox regression analysis. Similar results in non-small cell lung cancer, patients with high GHET1 expression had short overall survival time and high GHET1 expression was an independent unfavorable prognostic predictor for overall survival [11,12]. Moreover, Jin et al. showed that hepatocellular carcinoma cases with high GHET1 expression had poor prognosis and high GHET1 expression was an independent poor prognostic factor for overall survival [14]. Besides, there was a negative correlation between GHET1 expression and survival time in gastric cancer [10], breast cancer [15], esophageal squamous cell carcinoma [17], bladder cancer [19], and head and neck cancer [20]. Generally, high GHET1 expression is an unfavorable biomarker for most human cancers.

GHET1 has been suggested to function as oncogenic role in human cancer. However, the biological function of GHET1 was still unknown in cervical cancer. Based on the clinical results about GHET1 in cervical cancer, we guessed that GHET1 also functions as oncogenic lncRNA to regulate tumor cell proliferation, migration, and invasion in cervical cancer, like it in other cancers. In our results, we preliminarily found that knockdown of GHET1 expression markedly inhibited cell proliferation, migration, and invasion in cervical cancer, which was consistent with its function in other cancers. Moreover, several studies showed that GHET1 was involved in regulating epithelial-mesenchymal transition process in hepatocellular carcinoma [13], breast cancer [15], colorectal cancer [16], esophageal squamous cell carcinoma [17], bladder cancer [19], and osteosarcoma [21]. In our future research, we will further explore the biological function and molecular mechanism about GHET1 in cervical cancer.

In conclusion, GHET1 expression is significantly increased in cervical cancer tissues and cells, and associated with clinical progression and poor prognosis in patients with cervical cancer. Knockdown of GHET1 expression markedly inhibits cervical cancer cell proliferation, migration, and invasion.

## Abbreviations

AUC: area under the curve
CCK-8: cell counting kit-8
DMEM: dulbecco’s modified eagle medium
FBS: fetal bovine serum
GAPDH: glyceraldehyde-3-phosphate dehyfrogenase
GHET1: gastric carcinoma proliferation enhancing transcript 1
HPV: human papillomavirus
qRT-PCR: quantitative real time PCR
ROC: receiver operating characteristic

## References

[1] Bray F., Ferlay J., Soerjomataram I., Siegel R.L., Torre L.A. and Jemal A. (2018) Global cancer statistics 2018: GLOBOCAN estimates of incidence and mortality worldwide for 36 cancers in 185 countries. CA Cancer J. Clin. 68, 394–424 10.3322/caac.21492 30207593

[2] Siegel R.L., Miller K.D. and Jemal A. (2018) Cancer statistics, 2018. CA Cancer J. Clin. 68, 7–30 10.3322/caac.21442 29313949

[3] Castle P.E. and Maza M. (2016) Prophylactic HPV vaccination: past, present, and future. Epidemiol. Infect. 144, 449–468 10.1017/S0950268815002198 26429676

[4] Chen W., Zheng R., Baade P.D., Zhang S., Zeng H., Bray F. (2016) Cancer statistics in China, 2015. CA Cancer J. Clin. 66, 115–132 10.3322/caac.21338 26808342

[5] Chen W., Sun K., Zheng R., Zeng H., Zhang S., Xia C. (2018) Cancer incidence and mortality in China, 2014. Chin. J. Cancer Res. 30, 1–12 10.21147/j.issn.1000-9604.2018.01.01 29545714 PMC5842223

[6] Regalado Porras G.O., Chavez Nogueda J. and Poitevin Chacon A. (2018) Chemotherapy and molecular therapy in cervical cancer. Rep. Pract. Oncol. Radiother. 23, 533–539 10.1016/j.rpor.2018.09.002 30534017 PMC6277350

[7] Li H., Wu X. and Cheng X. (2016) Advances in diagnosis and treatment of metastatic cervical cancer. J. Gynecol. Oncol. 27, e43 10.3802/jgo.2016.27.e43 27171673 PMC4864519

[8] Sun W., Yang Y., Xu C., Xie Y. and Guo J. (2016) Roles of long noncoding RNAs in gastric cancer and their clinical applications. J. Cancer Res. Clin. Oncol. 142, 2231–2237 10.1007/s00432-016-2183-7 27246953 PMC11819183

[9] Li J., Jiang X., Li Z., Huang L., Zhou Y., Liu Y. (2019) Long noncoding RNA GHET1 in human cancer. Clin. Chim. Acta 488, 111–115 10.1016/j.cca.2018.11.007 30399371

[10] Yang F., Xue X., Zheng L., Bi J., Zhou Y., Zhi K. (2014) Long non-coding RNA GHET1 promotes gastric carcinoma cell proliferation by increasing c-Myc mRNA stability. FEBS J. 281, 802–813 10.1111/febs.12625 24397586

[11] Shen Q.M., Wang H.Y. and Xu S. (2018) LncRNA GHET1 predicts a poor prognosis of the patients with non-small cell lung cancer. Eur. Rev. Med. Pharmacol. Sci. 22, 2328–2333 29762836 10.26355/eurrev_201804_14823

[12] Guan Z.B., Cao Y.S., Li Y., Tong W.N. and Zhuo A.S. (2018) Knockdown of lncRNA GHET1 suppresses cell proliferation, invasion and LATS1/YAP pathway in non small cell lung cancer. Cancer Biomark. 21, 557–563 10.3233/CBM-170431 29286919 PMC13078292

[13] Ding G., Li W., Liu J., Zeng Y., Mao C., Kang Y. (2017) LncRNA GHET1 activated by H3K27 acetylation promotes cell tumorigenesis through regulating ATF1 in hepatocellular carcinoma. Biomed. Pharmacother. 94, 326–331 10.1016/j.biopha.2017.07.046 28772210

[14] Jin L., He Y., Tang S. and Huang S. (2018) LncRNA GHET1 predicts poor prognosis in hepatocellular carcinoma and promotes cell proliferation by silencing KLF2. J. Cell. Physiol. 233, 4726–4734 10.1002/jcp.26257 29139562

[15] Song R., Zhang J., Huang J. and Hai T. (2018) Long non-coding RNA GHET1 promotes human breast cancer cell proliferation, invasion and migration via affecting epithelial mesenchymal transition. Cancer Biomark. 22, 565–573 10.3233/CBM-181250 29843220 PMC13078473

[16] Zhou J., Li X., Wu M., Lin C., Guo Y. and Tian B. (2016) Knockdown of long noncoding RNA GHET1 inhibits cell proliferation and invasion of colorectal cancer. Oncol. Res. 23, 303–309 10.3727/096504016X14567549091305 PMC7838607 27131316

[17] Liu H., Zhen Q. and Fan Y. (2017) LncRNA GHET1 promotes esophageal squamous cell carcinoma cells proliferation and invasion via induction of EMT. Int. J. Biol. Markers 32, e403–e408 10.5301/ijbm.5000304 28983895

[18] Zhou H.Y., Zhu H., Wu X.Y., Chen X.D., Qiao Z.G., Ling X. (2017) Expression and clinical significance of long-non-coding RNA GHET1 in pancreatic cancer. Eur. Rev. Med. Pharmacol. Sci. 21, 5081–5088 29228419 10.26355/eurrev_201711_13822

[19] Li L.J., Zhu J.L., Bao W.S., Chen D.K., Huang W.W. and Weng Z.L. (2014) Long noncoding RNA GHET1 promotes the development of bladder cancer. Int. J. Clin. Exp. Pathol. 7, 7196–7205 25400817 PMC4230137

[20] Liu H. and Wu Y. (2018) Long non-coding RNA gastric carcinoma highly expressed transcript 1 promotes cell proliferation and invasion in human head and neck cancer. Oncol. Lett. 15, 6941–6946 29725422 10.3892/ol.2018.8185 PMC5920369

[21] Yang W., Shan Z., Zhou X., Peng L., Zhi C., Chai J. (2018) Knockdown of lncRNA GHET1 inhibits osteosarcoma cells proliferation, invasion, migration and EMT *in vitro* and *in vivo*. Cancer Biomark. 23, 589–601 10.3233/CBM-181863 30475755 PMC13078580

[22] Ni W., Luo L., Zuo P., Li R.P., Xu X.B., Wen F. (2018) lncRNA GHET1 down-regulation suppresses the cell activities of glioma. Cancer Biomark. 23, 9–22 10.3233/CBM-171002 30103301 PMC13078550

[23] Li T., Mo X., Fu L., Xiao B. and Guo J. (2016) Molecular mechanisms of long noncoding RNAs on gastric cancer. Oncotarget 7, 8601–8612 26788991 10.18632/oncotarget.6926 PMC4890990

[24] Huang H., Liao W., Zhu X., Liu H. and Cai L. (2017) Knockdown of long noncoding RNA GHET1 inhibits cell activation of gastric cancer. Biomed. Pharmacother. 92, 562–568 10.1016/j.biopha.2017.05.088 28577495

[25] Zhang X., Bo P., Liu L., Zhang X. and Li J. (2017) Overexpression of long non-coding RNA GHET1 promotes the development of multidrug resistance in gastric cancer cells. Biomed. Pharmacother. 92, 580–585 10.1016/j.biopha.2017.04.111 28578256

